# The role of nutrition-based index in predicting survival of breast cancer patients: A systematic review and meta-analysis

**DOI:** 10.1016/j.heliyon.2023.e23541

**Published:** 2023-12-11

**Authors:** Patricia Diana Prasetiyo, Bernard Agung Baskoro, Timotius Ivan Hariyanto

**Affiliations:** a Department of Pathology, Faculty of Medicine, Pelita Harapan University, Tangerang, Banten, 15811, Indonesia; b Division of Surgical Oncology, Department of Surgery, Faculty of Medicine, Pelita Harapan University, Karawaci, Tangerang, 15811, Indonesia; c Faculty of Medicine, Pelita Harapan University, Karawaci, Tangerang, 15811, Indonesia

**Keywords:** Nutrition index, Prognosis, Breast cancer, Neoplasm, Oncology

## Abstract

**Background:**

Prognostic nutritional index (PNI) and Controlling Nutritional Status (CONUT) are two model that incorporates the role of inflammation and nutrition factors to predict the progression of tumor. The primary objective of this investigation is to examine the ability of PNI and CONUT score for predicting the survival in breast cancer patients.

**Methods:**

A comprehensive search was conducted on the Cochrane Library, Scopus, Europe PMC, and Medline databases up until August 14th, 2023, utilizing a combination of relevant keywords. This review incorporates literature that examines the relationship between PNI, CONUT, and survival in breast cancer. We employed random-effect models to analyze the hazard ratio (HR) and present the outcomes together with their corresponding 95 % confidence intervals (CI).

**Results:**

A total of sixteen studies were incorporated. The results of our meta-analysis indicated that high PNI was associated with better overall survival (OS) (HR 0.38; 95%CI: 0.28–0.51, *p* < 0.00001, *I*^*2*^ = 32 %), but not disease-free survival (DFS) (HR 0.60; 95%CI: 0.33–1.10, *p* = 0.10, *I*^*2*^ = 78 %) than low PNI in breast cancer patients. Meta-analysis also indicated that high CONUT was associated with worse OS (HR 1.66; 95%CI: 1.21–2.28, *p* = 0.002, *I*^*2*^ = 78 %) and worse DFS (HR 2.09; 95%CI: 1.60–2.73, *p* < 0.00001, *I*^*2*^ = 41 %) in breast cancer patients.

**Conclusions:**

This study suggests the prognostic role of both PNI and CONUT score for predicting survival in breast cancer patients.

## 1 Introduction

Breast cancer is one of the most common types of cancer, especially in postmenopausal women [1]. Based on 2020 data, there were around 2.26 million new cases of breast cancer worldwide [1,2]. The anticipated trend indicates that the number of new cases is expected to rise to 2.7 million by the year 2030 [1,2]. Apart from having a high prevalence, breast cancer is also the second-leading cause of death in women after lung cancer, with a mortality rate of around 685 thousand cases in 2020, the majority of which come from countries in Asia and Africa [1,2]. Therefore, early detection and risk stratification are the main things to consider in efforts to reduce the burden of breast cancer.

Several prognostic models based on physical examination data and laboratory results have been developed to predict the progression and survival of cancer patients, including breast cancer [[3], [4], [5]]. Neutrophil-to-lymphocyte ratio (NLR), lymphocyte-to-monocyte ratio (LMR), c-reactive protein (CRP), and systemic immune-inflammation index (SII) are some disease markers or models that have been shown to be able to predict the prognosis of breast cancer [[3], [4], [5]]. These models are based on an understanding of the inflammatory conditions that play a role in the pathogenesis and progression of breast cancer [[3], [4], [5]]. In addition to inflammatory conditions, nutrition is also an important factor that can affect the course of breast cancer [6]. Previous studies have shown that the presence of anorexia or cachexia correlates with a poor prognosis, such as tumor development, metastases, and decreased response to chemotherapy in patients with cancer [6,7]. Considering this, we need a model that is easy to calculate, takes into account these two important factors, and can be used to predict the prognosis of cancer patients.

The prognostic nutritional index (PNI) is an indicator based on albumin and lymphocyte values that combines the important roles of both inflammatory processes and nutritional factors [8]. Although initially used to evaluate the nutritional status of patients undergoing surgery, recent studies have reported that PNI is also associated with response to therapy and survival rates in various cancers [8]. For example, a meta-analysis from 10 studies revealed that low PNI score was associated with worse overall survival (OS) and cancer specific survival (CSS), irrespective of region, TNM stage, and cut-off value used [8]. Apart from PNI, controlling nutritional status (CONUT) is also a simple tool based on albumin, total cholesterol, and total lymphocyte values that can be used for patient risk stratification [9]. Patients with high CONUT score (≥3) had significantly shorter OS than those with low CONUT scores [9]. Unfortunately, evidence regarding the use of PNI and CONUT as predictors of prognosis in patients with breast cancer is still scarce and conflicting. This study aims to evaluate the ability of both nutritional indexes, namely PNI and CONUT, to predict the survival of patients with breast cancer.

## 2 Materials and methods

### 2.1 Eligibility criteria

This systematic review and meta-analysis have been conducted in accordance with the guidelines outlined in the Preferred Reporting Items for Systematic Reviews and Meta-Analyses (PRISMA) statement [10]. We included all research that fulfilled the PECOS-based inclusion criteria as follows.

(1) Population = patients with the diagnosis of breast cancer of any stage or any type;
(2) Exposure and Control = have data regarding the comparison between high PNI vs. low PNI and high CONUT vs. low CONUT;
(3) Outcome = assessed the relationship between variables of interest (PNI and CONUT) with the overall survival (OS) and disease-free survival (DFS) in the form of hazard ratio (HR);
(4) Study Design = observational study (cohort, case-control, ataupun cross-sectional).

Meanwhile, the exclusion criteria were established in the following manner: (1) investigation focuses on the general cancer population without specific data on breast cancer; (2) cell-based or animal studies; (3) studies with incomplete data on the PNI or CONUT; (4) non-primary investigation; (5) research articles that are not accessible in their entirety or studies that have not undergone the process of publication.

### 2.2 Search strategy and study selection

A comprehensive review of the literature was performed, focusing specifically on papers written in the English language. The search encompassed a time frame up until August 14th, 2023, and was undertaken across four prominent worldwide databases: Medline, Scopus, Europe PMC, and the Cochrane Library. The search terms utilized for the literature review were as follows: “(breast cancer OR breast neoplasm OR breast malignancy OR breast mass OR breast tumor OR breast tumour OR breast adenoma OR breast carcinoma OR mammary cancer OR cancer of the breast OR carcinoma of breast OR neoplasm of breast) AND (prognostic nutritional index OR PNI OR controlling nutritional status OR CONUT)”. Supplementary Table 1 provides additional information pertaining to the search approach employed for each database. The initial search was conducted by two authors, and to ensure the search strategy's accuracy and reliability, third author independently reran the database search and verified the number of citations identified. The team also cross-checked the citations exported to the reference manager for consistency and completeness. To identify any additional relevant articles, citation tracking was performed by examining the references of the identified studies, tracking citations, and exploring related articles. Additionally, a follow-up search of gray literature sources was conducted. Titles and abstracts were screened independently by two authors, who excluded articles not relevant to the study. Full-text eligibility was conducted by the same two authors and the discrepancies were addressed in study judgments.

### 2.3 Data Extraction

Two reviewers independently retrieved essential data extracted from the eligible articles including characteristics of participants (i.e., age, breast cancer clinical stage, molecular subtype of breast cancer, receptor positivity, and treatment received) in addition to study characteristics (i.e., author last name, year of publication, country, study design, and number of participants). Prognostic Nutritional Index (PNI) was calculated by using the following formula: [(10 × serum albumin (g/dL)) + (0.005 × total lymphocyte count)]. The Controlling Nutritional Status (CONUT) score was calculated by incorporating data on the serum albumin (g/dL), total cholesterol (mg/dL), and total lymphocyte count (mm^3^).

### 2.4 Risk of bias assessment

The evaluation of potential bias in each study was conducted by two independent reviewers using standardized assessment tools. The Newcastle-Ottawa Scale (NOS) was employed to assess the quality of each observational study [11]. This scale incorporates evaluations about the selection of study participants, comparability between groups, and the outcomes of the studies [11]. The potential range of scores achievable by utilization of this instrument encompassed values from 0 to 9 [11]. In the context of research, studies attaining a total score of 7 or more were deemed to possess a commendable level of quality (good quality) [11].

### 2.5 Statistical analysis

The overall survival (OS) and disease-free survival (DFS) outcomes were calculated by using the Generic-Inverse Variance formula to obtain the hazard ratio (HR) along with the 95 % confidence interval (95 % CI). All of the data inputted in the analysis were multivariable-adjusted data of OS and DFS. The presence of diverse cancer stadium, molecular subtype, and treatment received as well as variable cut-off values for PNI and CONUT necessitated the consideration of a substantial amount of variability. To address this, random-effect models were employed. The I-squared (I^2^; Inconsistency) statistic was employed to quantify the heterogeneity among research, where values exceeding 50 % indicated a substantial or noteworthy level of heterogeneity [12]. Subgroup analysis of PNI variable based on country of origin and clinical stage of breast cancer would be performed. If the number of papers included in the meta-analysis exceeds 10, a funnel plot would be employed to evaluate the presence of publication bias. All analyses in this investigation were conducted using Review Manager 5.4, a software tool developed by the Cochrane Collaboration.

## 3 Results

### 3.1 Study selection and characteristics

**Fig. 1:**
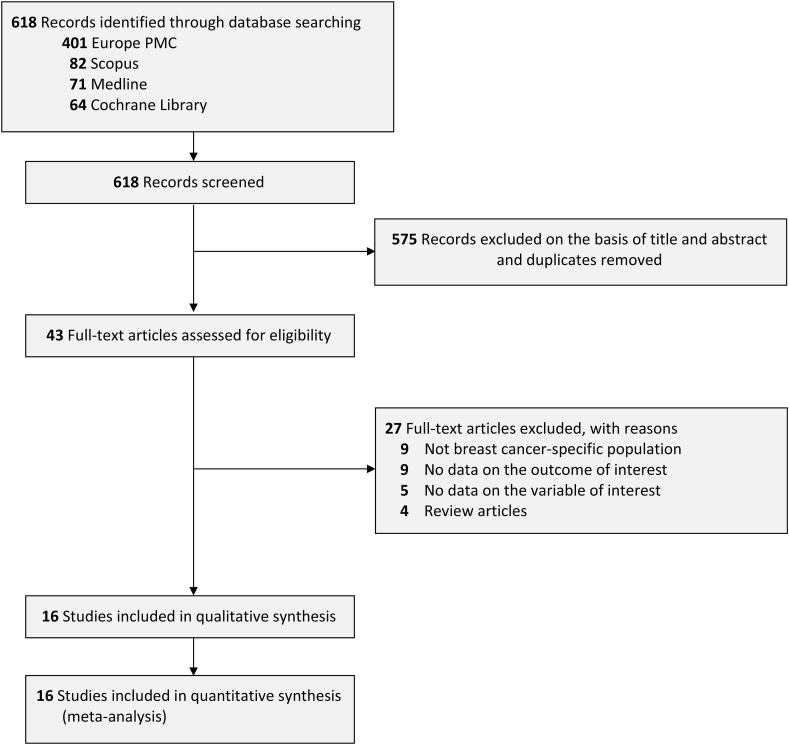
PRISMA diagram of the detailed process of selection of studies for inclusion in the systematic review and meta-analysis.

In this review we searched 4 databases; Europe PMC (n = 401), Scopus (n = 82), PubMed Medline (n = 71), and Cochrane Library (n = 64). A total of 618 citations were retrieved for screening. We removed 575 citations as they were found to be duplicates and not eligible based on title/abstracts screening. Of 43 records screened for full-text eligibility, 27 studies were excluded based on the following reasons: nine articles were not using breast cancer specific population, nine articles lacked data pertaining to the outcomes, five articles lacked data pertaining to the variable of interest, and four articles were omitted as they were review articles. Ultimately, the remaining 16 articles [[13], [14], [15], [16], [17], [18], [19], [20], [21], [22], [23], [24], [25], [26], [27], [28]] with a total of 7457 breast cancer patients were included in the final analysis (Fig. 1). Almost all of the included studies had retrospective cohort design with only 1 study that had prospective cohort design. Out of 16 studies, 11 studies came from China with the remaining five studies came from Japan. The cut-off values used to define high and low PNI were varied across the included studies, ranging from 48.7 to 55. Unlike PNI, most included studies used the value of 3 to differentiate high and low CONUT score. Data regarding breast cancer stadium, their molecular subtype, and treatment received can be seen in Table 1 where a comprehensive overview of the characteristics of each study included in this analysis was provided.

**Table 1:** Characteristics of included studies.

Study	Sample size	Country	Design	Cutoff	Cancer stage	Molecular subtype	Cancer receptor			Age (years)	Treatment (%)
							ER+	PR+	HER2+		
Chen L et al. [13] 2021	785	China	RC	≥51 (PNI)	- I: 11.7 % -II: 48.6 % -III: 39.6 %	-Luminal A: 7.9 % -Luminal B/HER2+: 12.4 % -Luminal B/HER2-: 41.4 % -HER2 enriched: 16.4 % -Triple negative: 21.7 %	62.3 %	59.8 %	29 %	47.1	-Surgical resection: 100 % -Chemotherapy: 65.7 % -Radiotherapy: 75 % -Endocrine therapy: 61.5 % -Targeted therapy: 25.7 %
Huang Z et al. [14] 2020	1367	China	RC	≥3 (CONUT)	-I: 23.7 % -II: 50.8 % -III: 23.6 % -IV: 2 %	-Luminal A: 25.8 % -Luminal B/HER2+: 14.8 % -Luminal B/HER2-: 29.6 % -HER2 enriched: 13.5 % -Triple negative: 15.9 %	68.8 %	60.9 %	28.2 %	48.3	-Surgical resection: 100 %
Hua X et al. [15] 2020	380	China	RC	≥52 (PNI)	-I: 33.9 % -II: 66.1 %	Not reported	71.1 %	65.3 %	28.9 %	47.2	-Mastectomy: 100 % -Chemotherapy: 87.1 % -Radiotherapy: 28.4 % -Endocrine therapy: 48.9 %
Li W et al. [16] 2020	861	China	RC	≥3 (CONUT)	Not reported	-Luminal A: 25.9 % -Luminal B: 47.3 % -HER2 enriched: 15.7 % -Triple negative: 11.1 %	62.5 %	46.2 %	23 %	55.8	-Mastectomy: 79.9 % -BCS: 20.1 % -Chemotherapy: 70.4 % -Radiotherapy: 45.6 % -Hormonal therapy: 76.1 %
Li Y et al. [17] 2023	697	China	RC	≥3 (CONUT)	-I: 38.6 % -II: 43.5 % -III: 17.9 %	-Luminal A: 40.5 % -Luminal B: 42.6 % -Triple negative: 16.9 %	82.8 %	78.2 %	0 %	52	-Mastectomy: 93.7 % -BCS: 6.3 % -Chemotherapy: 82.4 % -Radiotherapy: 25.1 % -Endocrine therapy: 76.5 %
Mohri T et al. [18] 2016	212	Japan	RC	≥52.4 (PNI)	-I: 64 % -II: 27 % -III: 9 %	Not reported	79 %	68 %	18 %	65.7	-Surgical resection: 100 %
Oba T et al. [19] 2020	191	Japan	RC	NR	-I: 0.5 % -II: 61.8 % -III: 37.7 % -IV: 0 %	-Luminal: 56 % -Luminal HER2: 19.4 % -HER2 enriched: 12.6 % -Triple negative: 12 %	NR	NR	NR	51.2	-Mastectomy: 67.1 % -Partial resection: 32.9 %
Oba T et al. [20] 2021	60	Japan	RC	NR	-IV: 100 %	Not reported	NR	NR	NR	NR	-Chemotherapy: 100 %
Sun L et al. [21] 2023	135	China	RC	>51 (PNI)	Not reported	Not reported	83 %	77 %	19.3 %	53	-Surgical resection: 100 %
Wang Y et al. [22] 2019	202	China	RC	≥55 (PNI)	Not reported	Not reported	68.8 %	75.2 %	41.1 %	NR	-Surgical resection: 100 %
Xu T et al. [23] 2022	508	China	RC	>53 (PNI)	−0: 7.3 % -I: 20.1 % -II: 51.7 % -III: 19.7 % -IV: 0.2 %	Not reported	70.3 %	59.6 %	68.3 %	49.7	-Total mastectomy: 37.4 % -Modified radical mastectomy: 49.02 % -BCS: 12.01 % -Chemotherapy: 72.4 % -Radiotherapy: 30.1 % -Endocrine therapy: 68.5 % -Targeted therapy: 9.1 %
Yamamoto S et al. [24] 2022	110	Japan	RC	≥55 (PNI)	-IV: 100 %	Not reported	68.2 %	47.3 %	NR	NR	-Chemotherapy: 100 % -Endocrine therapy: 51.8 %
Yamanouchi K et al. [25] 2023	35	Japan	RC	>48.7 (PNI)	-IV: 100 %	-Luminal: 57.1 % -HER2: 17.1 % -Triple negative: 25.7 %	NR	NR	NR	64.7	-Systemic therapy: 100 %
Yang Z et al. [26] 2014	382	China	RC	≥48.7 (PNI)	Not reported	-Triple negative: 100 %	0 %	0 %	0 %	50.1	-Surgical resection: 100 % -Chemotherapy: 89.2 % -Radiotherapy: 18.3 %
Zhang X et al. [27] 2023	1151	China	PC	≥3 (CONUT)	-I: 17.8 % -II: 33.8 % -III: 17.9 % -IV: 30.5 %	Not reported	NR	NR	NR	52.5	Not reported
Zhu M et al. [28] 2022	381	China	RC	≥1 (CONUT)	Not reported	-Luminal A: 7.6 % -Luminal B/HER2+: 10.2 % -Luminal B/HER2-: 45.6 % -HER2 enriched: 17.3 % -Triple negative: 19.2 %	54.8 %	38.5 %	28.1 %	50	-Mastectomy: 93.2 % -BCS: 6.8 % -Chemotherapy: 63.5 % -Radiotherapy: 79.2 % -Endocrine therapy: 58 % -Targeted therapy: 18.9 %

BCS = breast conserving surgery; CONUT = controlling nutritional status; ER = estrogen receptor; HER2 = human epidermal growth factor receptor 2; NR = not reported; PC = prospective cohort; PNI = prognostic nutritional index; PR = progesterone receptor; RC = retrospective cohort.

### 3.2 Quality of study assessment

All cohort studies included in this study demonstrated high quality as assessed by the Newcastle Ottawa Scale (NOS), with values ranging from 7 to 8 (Table 2). All studies were considered suitable for inclusion in the meta-analysis.

**Table 2:** Newcastle-Ottawa quality assessment of observational studies.

First author, year	Study design	Selection^a^	Comparability^b^	Outcome^c^	Total score	Result
Chen L et al. [13] 2021	Cohort	***	**	***	8	Good
Huang Z et al. [14] 2020	Cohort	***	**	***	8	Good
Hua X et al. [15] 2020	Cohort	***	**	***	8	Good
Li W et al. [16] 2020	Cohort	***	**	***	8	Good
Li Y et al. [17] 2023	Cohort	***	**	***	8	Good
Mohri T et al. [18] 2016	Cohort	***	**	**	7	Good
Oba T et al. [19] 2020	Cohort	***	**	**	7	Good
Oba T et al. [20] 2021	Cohort	***	**	**	7	Good
Sun L et al. [21] 2023	Cohort	***	**	***	8	Good
Wang Y et al. [22] 2019	Cohort	***	**	***	8	Good
Xu T et al. [23] 2022	Cohort	***	**	***	8	Good
Yamamoto S et al. [24] 2022	Cohort	***	**	**	7	Good
Yamanouchi K et al. [25] 2023	Cohort	***	**	**	7	Good
Yang Z et al. [26] 2014	Cohort	***	**	***	8	Good
Zhang X et al. [27] 2023	Cohort	***	**	**	7	Good
Zhu M et al. [28] 2022	Cohort	***	**	***	8	Good

a (1) is the case definition adequate; (2) representativeness of the cases; (3) selection of controls; (4) definition of controls.

b (1) comparability of cases and controls on the basis of design or analysis, (maximum two stars).

c (1) ascertainment of exposure; (2) same method of ascertainment for cases and controls; (3) non-response rate.

### 3.3 Prognostic nutritional index (PNI)

#### 3.3.1 Overall survival (OS)

**Fig. 2:**
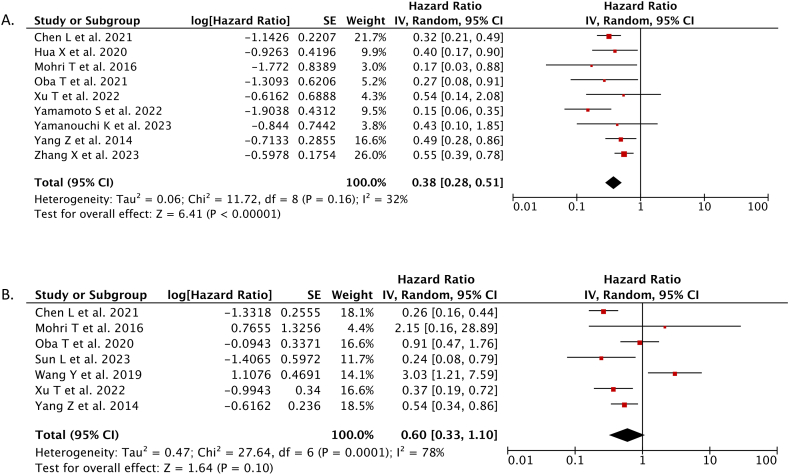
Forest plot that demonstrates the comparison between high prognostic nutritional index (PNI) score and low PNI score for predicting the overall survival (A) and disease-free survival (DFS) in breast cancer patients.

There were 9 studies which reported the adjusted overall survival of breast cancer patients based on PNI score. The meta-analysis from these studies showed high PNI score was independently associated with better overall survival (OS) in breast cancer patients than low PNI score (HR 0.38; 95%CI: 0.28–0.51, *p* < 0.00001, *I*^*2*^ = 32 %, random-effect model) (Fig. 2A).

#### 3.3.2 Disease-free survival (DFS)

There were 7 studies which reported the adjusted disease-free survival (DFS) of breast cancer patients based on PNI score. The meta-analysis from these studies showed the disease-free survival (DFS) did not differ significantly between those who had high PNI score and those who had low PNI score (HR 0.60; 95%CI: 0.33–1.10, *p* = 0.10, *I*^*2*^ = 78 %, random-effect model) (Fig. 2B).

### 3.4 Controlling nutritional status (CONUT)

#### 3.4.1 Overall survival (OS)

**Fig. 3:**
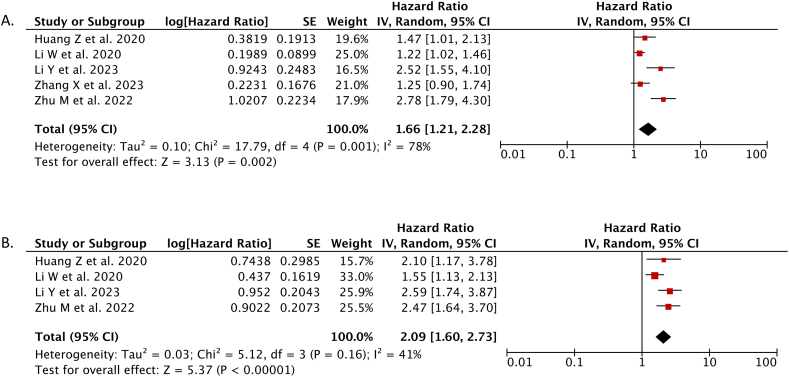
Forest plot that demonstrates the comparison between high controlling nutritional status (CONUT) score and low CONUT score for predicting the overall survival (A) and disease-free survival (DFS) in breast cancer patients.

Our meta-analysis from 5 studies showed high CONUT score was independently associated with worse overall survival (OS) in breast cancer patients than low CONUT score (HR 1.66; 95%CI: 1.21–2.28, *p* = 0.002, *I*^*2*^ = 78 %, random-effect model) (Fig. 3A).

#### 3.4.2 Disease-free survival (DFS)

Our meta-analysis from 4 studies showed high CONUT score was independently associated with worse disease-free survival (DFS) in breast cancer patients than low CONUT score (HR 2.09; 95%CI: 1.60–2.73, *p* < 0.00001, *I*^*2*^ = 41 %, random-effect model) (Fig. 3B).

### 3.5 Subgroup analysis of prognostic nutritional index (PNI)

#### 3.5.1 Overall survival (OS)

We performed subgroup analysis on the overall survival outcome (OS) of prognostic nutritional index (PNI) score based on country of origin and clinical stage of breast cancer patients. Subgroup analysis based on the country of origin revealed a lower HR for studies from Japan (HR 0.21; 95%CI: 0.11–0.37, *p* < 0.00001, *I*^*2*^ = 0 %, random-effect model) than studies from China (HR 0.45; 95%CI: 0.36–0.57, *p* < 0.00001, *I*^*2*^ = 0 %, random-effect model), but both still showed significant results (Supplementary Fig. 1). Meanwhile, subgroup analysis based on clinical stage of breast cancer showed a lower HR for studies with all grade IV breast cancer patients (HR 0.21; 95%CI: 0.11–0.40, *p* < 0.00001, *I*^*2*^ = 0 %, random-effect model) than studies with majority of grade I-III breast cancer patients (HR 0.44; 95%CI: 0.35–0.56, *p* < 0.00001, *I*^*2*^ = 6 %, random-effect model), but both still showed significant results (Supplementary Fig. 2).

#### 3.5.2 Disease-free survival (DFS)

We also performed subgroup analysis on the disease-free survival (DFS) of prognostic nutritional index (PNI) score based on country of origin. Subgroup analysis based on the country of origin revealed a lower HR for studies from China (HR 0.51; 95%CI: 0.25–1.05, *p* = 0.07, *I*^*2*^ = 82 %, random-effect model) than studies from Japan (HR 0.96; 95%CI: 0.51–1.82, *p* = 0.90, *I*^*2*^ = 0 %, random-effect model), but both still showed non-significant results (Supplementary Fig. 3). Subgroup analysis based on the clinical stage of breast cancer cannot be performed due to no studies with all stage IV breast cancer that reported the DFS outcome.

### 3.6 Publication bias

Funnel plot analysis was employed to assess publication bias. The present investigation did not perform the assessment of publication bias due to the limited number of research included (less than 10 studies) in each outcomes of interest. Consequently, the evaluation of publication bias lacks the same level of robustness as when there are more than 10 studies available for analysis [29,30].

## 4 Discussion

The findings of our meta-analysis indicate that both prognostic nutritional index (PNI) and Controlling Nutritional Status (CONUT) can be used to predict the prognosis of breast cancer patients where high PNI score but low CONUT score were associated with better overall survival (OS) from breast cancer. CONUT score can also be used to evaluate the disease-free survival (DFS) of breast cancer where high CONUT indicate worse DFS, while PNI cannot be used for this purpose. Subgroup analysis based on country of origin and clinical stage of breast cancer showed stronger association between high PNI and better OS in studies from Japan with all clinical stage IV breast cancer participants.

There are several things that can explain why the PNI score, which consists of serum albumin and total lymphocyte count, can be used to predict the prognosis of cancer, including breast cancer. Serum albumin can provide a simple picture of the function of visceral proteins [31,32]. Cancer is characterized by an accelerated proliferation of malignant cells, which is accompanied by an elevated metabolic rate [33]. Consequently, there is an augmented demand for nutritional supply and a corresponding need for increased energy generation [33]. Within the context of the tumor microenvironment, it has been observed that the tumor exhibits a rapid uptake of albumin as a compensatory response to the relatively deficient availability of amino acids [33]. This biological mechanism facilitates the tumor's ability to satisfy its elevated metabolic demands for rapid proliferation, leading to a decline in serum albumin concentrations [33]. In the context of cancer, a decrease in appetite might also be observed as a consequence of tumor chemotherapy or radiotherapy, leading to a reduction in protein consumption and hindered synthesis [34]. The occurrence of tissue damage and inflammation has the potential to expedite this catabolic process, resulting in a reduction in plasma albumin levels [34]. Simultaneously, tumor cells may release cytokines, such as IL-6, which impede the formation of albumin in hepatocytes [35]. The reduction in albumin production in cancer has been supported by an in-vivo investigation, wherein mice with tumors exhibited hypoalbuminemia, mostly attributed to a decline in the abundance of liver albumin mRNA [36,37]. The reduction in albumin synthesis will lead to an elevation in TNF-induced micro-vessel permeability, resulting in a decline in serum albumin concentrations [35,38]. Additionally, a prior investigation has indicated that malnutrition among cancer patients can contribute to a reduction in the synthesis rate of serum albumin, hastening its breakdown and thus leading to hypoproteinemia [38]. Therefore, in the initial stages of cancer, there is minimal or negligible hypoalbuminemia [38]. However, as the disease advances, there is a substantial decrease in albumin levels, which can effectively act as prognostic indicators for cancer [38].

Meanwhile, the measurement of lymphocyte count in the peripheral blood has emerged as a significant indicator in the prevention of tumor progression in various types of malignancies, as it facilitates the activation of the host immune system [39,40]. One example of cellular activity in the immune system involves CD8^+^ T cells, which play a significant role in immune surveillance and the body's response to tumors [41,42]. These cells achieve this by releasing various cytokines, including perforin and granzyme B [41,42]. CD4^+^ T-helper cells have the potential to enhance anti-tumor immune responses by secreting cytokines, such as IFN-γ [43]. 10.13039/100014337Furthermore, the fall in lymphocyte levels in the peripheral circulation would result in a reduction of these cells infiltrating tumors, so weakening the response to cytotoxic treatment and ultimately diminishing the overall survival rate of individuals with cancer [39,40]. The present discovery has been supported by in-vivo investigations conducted on animals with mammary tumors, wherein it was seen that the count of lymphocytes declines in conjunction with the advancement of cancer, leading to a diminished rate of survival [44]. When considering the collective evidence, it can be shown that a reduction in albumin or lymphocyte count in the peripheral blood is associated with the onset and advancement of tumors. Therefore, a decreased prognostic nutritional index (PNI) may provide benefits in terms of tumor cell survival and invasion, ultimately leading to a more unfavorable prognosis.

The findings of our meta-analysis align with the recent meta-analysis conducted by Hu G et al. [45] in 2023. In their meta-analysis, Hu G et al. [45] demonstrated that low PNI was associated with worse OS, but not DFS in patients with breast cancer. Nonetheless, there are some fundamental differences between our meta-analysis and the previous study by Hu G et al. [45].

First, Hu G et al. [45] conducted an earlier meta-analysis that included a total of 8 studies, with information on 6 studies that focused on OS analysis and 5 studies that focused on DFS analysis. Of these 8 studies, there was 1 study by Soberanis-Pina PD et al. [46], which was published in the form of an abstract. Although listed in the table of characteristics of the included studies, the study by Soberanis-Pina PD et al. [46] was not discussed further at all nor included in the meta-analysis. There is no clear reason why one study in the form of an abstract was still included. Abstracts are generally not recommended for inclusion in the meta-analysis unless the evidence is scarce and conflicting [47,48]. The reason is that the data presented in the Abstracts is very limited, both in the Methods and Results sections, so it will be difficult to assess the quality or risk of bias of the study [47,48]. Therefore, we have listed abstract-only articles as one of the exclusion criteria for this study. Our current meta-analysis also includes more studies, namely a total of 16 full-text studies, twelve of which specifically discuss the relationship between PNI, OS, and DFS. With more included studies, of course, it will be able to add to the strength of our evidence.

Second, there are doubts about the validity of the data included in the analysis of an earlier investigation by Hu G et al. [45] They incorporate data from the study by Oba T et al. [19] (2020) into a meta-analysis regarding overall survival (OS) and disease-free survival (DFS). In fact, when examined further, the study by Oba T et al. [19], which was published in 2020, only attaches data regarding DFS but does not discuss overall survival (OS) at all, both in the Methods and Results sections. In addition to DFS, another outcome presented in the study by Oba T et al. [19] in 2020 was only disease-specific survival (DSS), which is of course different from overall survival (OS). Data regarding OS can only be found in a newer study by the same author, which was published in 2021 in a different journal [20]. Disease-specific survival (DSS) is the duration from the date of diagnosis until death due to a specific disease (in this case, breast cancer) [49]. Meanwhile, overall survival (OS) is the duration from the date of diagnosis to death or last follow-up, with no restriction on the cause of death [49]. Therefore, DSS and OS cannot be used interchangeably and are two different entities. It is not known exactly where Hu G et al. [45] obtained data regarding OS in the 2020 study by Oba T et al. [19] On the other hand, our current meta-analysis only includes data according to what is stated in published articles and in their supplementary materials, so that we can guarantee the validity of the data used in the analysis.

Third, the earlier meta-analysis by Hu G et al. [45] only analyzed one type of variable as exposure, namely the Prognostic Nutritional Index (PNI). Meanwhile, our current meta-analysis does not only discuss PNI but also Controlling Nutrition Status (CONUT) as another exposure variable that can predict the prognosis of breast cancer. From the analysis using a total of 5 studies, we managed to show that the CONUT score can also be used to predict overall survival (OS) and disease-free survival (DFS) in breast cancer patients.

The present investigation is not devoid of limitations. All of the research covered in this study was conducted in Asian countries, namely China and Japan. Consequently, the generalizability of these findings may be constrained, particularly in relation to populations that are not of Asian descent. Second, the majority of studies included in the analysis only involved a small number of participants, with only two studies involving more than 1000 breast cancer patients. Third, the cut-off values used to define high and low PNI scores varied relatively between the included studies, causing heterogeneity in the analysis, especially in the DFS outcome.

## 5 Conclusion

The findings of our systematic review and meta-analysis indicate a potential ability of both Prognostic Nutritional Index (PNI) and Controlling Nutritional Status (CONUT) as prognostic marker of overall survival (OS) in breast cancer patients where high PNI and low CONUT were associated with better OS. Furthermore, low CONUT score was also associated with worse disease-free survival (DFS) in breast cancer patients. These nutrition-based scoring systems can be used as risk stratification tools to categorize breast cancer patients that may be at high risk of mortality, so that earlier and more comprehensive treatment can be provided to the patients. Nonetheless, additional meticulously planned investigations, especially from outside Asian countries, are still needed to validate the findings of our study.

## Funding

None.

## Ethics approval

This is a systematic review and meta-analysis study. The Faculty of Medicine, Pelita Harapan University Research Ethics Committee has confirmed that no ethical approval is required.

## Consent to participate

Not applicable.

## Consent to publish

Not applicable.

## Data availability statement

Data included in article/supp. material/referenced in article.

## CRediT authorship contribution statement

**Patricia Diana Prasetiyo:** Writing – review & editing, Writing – original draft, Visualization, Validation, Supervision, Software, Resources, Project administration, Methodology, Investigation, Funding acquisition, Formal analysis, Data curation, Conceptualization. **Bernard Agung Baskoro:** Writing – review & editing, Writing – original draft, Visualization, Validation, Supervision, Software, Resources, Project administration, Methodology, Investigation, Funding acquisition, Formal analysis, Data curation, Conceptualization. **Timotius Ivan Hariyanto:** Writing – review & editing, Writing – original draft, Visualization, Validation, Supervision, Software, Resources, Project administration, Methodology, Investigation, Funding acquisition, Formal analysis, Data curation, Conceptualization.

## Declaration of competing interest

The authors declare that they have no known competing financial interests or personal relationships that could have appeared to influence the work reported in this paper.
