# Supplementary material for: The role of nutrition-based index in predicting survival of breast cancer patients: A systematic review and meta-analysis
Source: Heliyon. 2023 Dec 11;10(1):e23541. doi: 10.1016/j.heliyon.2023.e23541 (PMC10758813; doi:10.1016/j.heliyon.2023.e23541)
Supplement: Multimedia component 1 [file mmc1.docx]

**Supplementary Table 1. Literature search strategy**

**1A. Medline Search String:**

“(breast cancer OR breast neoplasm OR breast malignancy OR breast mass OR breast tumor OR breast tumour OR breast adenoma OR breast carcinoma OR mammary cancer OR cancer of the breast OR carcinoma of breast OR neoplasm of breast) AND (prognostic nutritional index OR PNI OR controlling nutritional status OR CONUT)”

**1B. Example Scopus and Cochrane Library Search Strategy:**

1. breast cancer

2. breast neoplasm

3. breast malignancy

4. breast mass

5. breast tumor

6. breast tumour

7. breast adenoma

8. breast carcinoma

9. mammary cancer

10. cancer of the breast

11. carcinoma of breast

12. neoplasm of breast

13. prognostic nutritional index

14. PNI

15. controlling nutritional status

16. CONUT

17. 1 or 2 or 3 or 4 or 5 or 6 or 7 or 8 or 9 or 10 or 11 or 12

18. 13 or 14 or 15 or 16

19. 17 and 18

Supplementary Figure 1. Subgroup analysis of the comparison between high prognostic nutritional index (PNI) and low PNI on the overall survival (OS) outcome based on the country of origin.

Supplementary Figure 2. Subgroup analysis of the comparison between high prognostic nutritional index (PNI) and low PNI on the overall survival (OS) outcome based on the clinical stage of breast cancer patients.

Supplementary Figure 3. Subgroup analysis of the comparison between high prognostic nutritional index (PNI) and low PNI on the disease-free survival (DFS) outcome based on the country of origin.
